# Identification and epidemiological analysis of *Perostrongylus falciformis* infestation in Irish badgers

**DOI:** 10.1186/s13620-019-0144-6

**Published:** 2019-07-09

**Authors:** Jennifer OC. Byrne, Andrew W. Byrne, Annetta Zintl, Karolina Jankowska, Emmanuel Coulange, Theo de Waal, Grainne McCarthy, James O’Keeffe, Inger S. Hamnes, Ursula Fogarty

**Affiliations:** 1 Irish Equine Centre, Johnstown, Naas, County Kildare Ireland; 2 Veterinary Sciences Division, Agri-Food and Biosciences Institute (AFBI), Stoney Road, Belfast, BT4 3SD Northern Ireland; 3 UCD Veterinary Sciences Centre, University College Dublin, Dublin, Ireland; 4 Paul Sabatier University, Toulouse, France; 5 Department of Agriculture, Food and the Marine (DAFM), Dublin, Republic of Ireland; 6 Section of Parasitology, Norwegian Veterinary Institute, Sentrum, NO-0106 Oslo, Norway

**Keywords:** Badger, *Meles meles*, Lungworm, *Perostrongylus falciformis*, Ireland

## Abstract

**Background:**

The lungworm, Perostrongylus *falciformis* (fomerly known as *Aelurostrongylus falciformis*) has been identified in badgers (*Meles meles*) in Britain, the Russian Federation, Italy, Norway, Poland, Ukraine, Bosnia Herzegovina and Romania, while *Aelurostrongylus pridhami* has been reported from badgers in Spain.

**Results:**

Pulmonary tissue from 1580 Irish badgers was examined and an estimated prevalence of 32.09% (95% CI: 29.79–34.45%) of this parasite was detected. Confirmation of its occurrence was made by PCR analysis on a subset of the population.

**Conclusion:**

Infestation was widely distributed throughout the Republic of Ireland, with a trend towards higher infestation risk in western versus eastern counties. In addition males were at a higher risk of infestation than females and juveniles were at a significantly higher risk than adult badgers.

## Background

Irish badgers are now classified with their European counterpart as *Meles meles* and are recognised as distinct from those found in eastern Eurasia, Japan and Crete [1]. Recent population and landscape genetic studies on the Irish badger have shown that contemporary populations have links to present-day Iberian and Scandinavian badger populations particularly in the west of Ireland, with badgers on the north-east and eastern side of the island more closely related to those in Great Britain and continental Europe [2–4]. Badgers are infected with a variety of parasites including nematodes (*Uncinaria*, *Capillaria* and *Strongyloides* spp), cestodes (*Taenia* and *Mesocestoides* spp) and trematodes [5, 6]. A lungworm *Perostrongylus falciformis* was first described in German badgers by Schlegel in 1933 [7]. Following several reclassifications, this parasite subsequently became known in the literature as *Aelurostrongylus falciformis,* and was included in the same genus with *Aelurostrongylus abstrusus, Aelurostrongylus pridhami* and *Aelurostrongylus fengi* [8]. Recent molecular phylogenetic and morphological studies by Deak et al. [8] have reconfirmed the classification as *Perostrongylus* and have shown that *A. abstrusus* is distinct. *P. falciformis* has been identified in badgers in Britain [5], the Russian Federation [9], Italy [10], Norway [11], Poland [12], Ukraine [13], Bosnia Herzegovina [14] and Romania [8] while the species *A. pridhami* has been reported from badgers in Spain [15]. In contrast a survey of helminth infestations of the badger carried out in southwest Portugal failed to identify this parasite [16].

*P. falicformis* has an indirect life cycle with slugs and terrestrial snails serving as intermediate hosts [8]. Stage 1 (L1) larvae passed in the faeces of infected badgers penetrate the foot of the snail intermediate host where they develop to L3 larvae. Following ingestion by the definitive host, the parasite matures to the adult stage in the alveoli, alveolar ducts and terminal bronchioles. Thin-shelled eggs produced by the female worm hatch more or less immediately releasing L1 larvae which are coughed-up, swallowed and shed via the faeces. As with many wildlife parasites, infections with *P. falciformis* appear to be generally of low pathogenicity although rare severe and even fatal cases have been reported [8].

In 1974 badgers were identified as a wildlife host for bovine tuberculosis (bTB) in Ireland, a fact that has stimulated extensive research into the biology and ecology of this mammal [1, 17, 18]. Recent suggestions that helminth co-infecions may affect the host’s immune response to concurrent TB infections [19] have further increased scientific interest in parasitic worms infecting badgers, particularly those that inhabit the same site [17]. The culling of Irish badgers as part of the national bTB eradication programme [20] has provided a unique opportunity to monitor *P. falciformis* in a large sample of its wildlife host. This paper confirms the presence of *P. falciformis* in Irish badgers, and presents epidemiological analysis of this lungworm infestation in Irish badgers over the period 1995–2017.

## Materials & methods

### Surveillance samples

The badgers examined were those captured under a bovine TB eradication programme between 1995 and 2017, excluding 2010. These activities were coordinated through the National District Veterinary Offices organised on a county basis. Date, geographical location, age and sex of the badgers were recorded at the time of capture. Badgers were transported to the post mortem facility within 36 h of death, where they were stored at 0–4 °C until post mortem examination. The nature of the post mortem examination and sample collection were dictated by the requirements of the bTB eradication scheme rather than by an investigation into the occurrence/prevalence of lung- or heartworms specifically. There was no systematic bias in terms of geography, age or sex in the selection of badgers contributing to the dataset. However, lungs showing small nodular lesions on gross examination, primarily in the dorsal areas of the lung lobes were preferentially sampled because of the possibility they could be *Mycobacterium bovis* lesions.

### Identification and detection of *P. falciformis* in the badger population using histopathology

One thousand, five hundred and-eighty badger pulmonary tissue samples were fixed in 10% formalin, routinely processed and stained with hemotoxylin and eosin. The presence or absence of adult pulmonary parasites in the distal airways was recorded. Morphological characterisation was confirmed by experts from the Norwegian Veterinary Institute (Inger Sofie Hamnes) and the Natural History Museum, London (Eileen Harris). Histopathological examination of the lungs was carried out independently of the badger population demographics. The latter were subsequently retrieved from archival records and digitized within Microsoft Excel. With the continual reduction in the prevalence of *M. bovis* infection in badgers over the study period [21] the number of pulmonary tissue samples collected decreased particularly post 2007 (Table 1).

**Table 1:** The number of pulmonary tissue samples collected from badgers over the study period. Samples were not collected in 2010

Year	1995	1996	1997	1998	1999	2000	2001	2002
No. of Samples	2	109	34	46	47	41	100	151
Year	2003	2004	2005	2006	2007	2008	2009	2010
No. of Samples	192	157	124	338	131	2	2	n/a
Year	2011	2012	2013	2014	2015	2016	2017	
No. of Samples	9	12	5	6	4	13	55	

### Molecular analysis for the detection and identification of badger lungworm

PCR analysis for molecular detection and identification of lung- and heartworms was carried out on a total of 15 badgers, captured in counties Carlow (1), Kildare (4), Kilkenny (1), Laois (1), Offaly (1), Tipperary (4) and Wexford (1) in 2017. For each animal two samples were analysed. One of these was collected by injecting 10 ml of tap water into the upright pluck using a syringe, the other by reverse flushing of the left ventricle, pulmonary veins and pulmonary capillaries. Following centrifugation (1330 g) and DNA extraction using the QIAamp DNA mini kit, all samples were amplified using three nested PCR assays designed to amplify different regions of the nuclear rRNA genes and internal transcribed spacers. Primer sequences are listed in Table 2. All PCR assays were performed at an annealing temperature of 55 °C under standard conditions. Amplicons were purified (High Pure PCR Product Purification Kit) and sequenced in both directions using the internal PCR primers (Eurofins Genomics).

**Table 2:** Primer sequences used to amplify fragments of the rRNA genes and internal transcribed spacers

Locus (size)	Primer	Sequence	Ref
18S (700 bp)	External	Fw: 5′ AAAGATTAAGCCATGCA 3′ Rev.: 5’GCAGGTTCACCTACAGAT 3’	[22]
	Nested	Fw: 5’ CGGCTCATTAGAGCAGATGTC 3′ Rev.: 5′ TCCTCTTTTATTATTCCATGATCG 3’	This study
ITS2 (548 bp)	External	Fw: 5’ TTTGAACGCATAGCGTCGT 3′ Rev.: 5′ TTAGTTTCTTTTCCTCCGCT 3’	This study [23]
	Hemi-nested	Fw: 5’ ACGTCTGGTTCAGGGTTGTT 3′ Rev.: 5′ TTAGTTTCTTTTCCTCCGCT 3’	[23]
Connecting fragment (1953 bp)	External	Fw: 5’ GCCTTTGGCGTTAATCACTG 3′ Rev.: 5′ TCACGATCATCAATCCATCG 3’	This study
	Nested	Fw: 5’ AGAACAAGCGTTTGCTTGAA3′ Rev.: 5′ GTATGACCAACAGCCGAACA3’	This study

### Data analysis and modelling

All data manipulation and analysis was undertaken within Stata 14 SE. Maps were created using ArcGIS 10.3.

All lungworm results were used to assess prevalence and the overall temporal trends. Prevalence was presented with 95% CI estimated using exact binomial CI. The outcome for each badger was modelled in a binary logit model with year fitted as i. categorical variable and ii. as a continuous linear trend. Where sample sizes were too small to estimate 95% CI for a given year (e.g. 2015 there were four observations, all were negative), these point and CI estimates were derived from an exact binomial distribution.

Univariable and multivariable modelling was undertaken on records where all covariate data were available. Covariate data included the county (location) where the animal was captured (*n* = 26), age-class (adult or juvenile), season (with winter: December, January, February; spring: March, April, May; summer: June, July, August and autumn: September, October, November) and sex of the animal (male/female). Logistic regression univariable models were used to investigate unadjusted relationships between infection status and each covariate respectively. A multivariable model was then developed using all variables as potential predictors of infection risk. We also investigated two-way interaction terms. Competing models were assessed using Akaike’s information criterion (AIC). The alpha for significance was set to 0.05 throughout. A Hosmer-Lemeshow test was used to assess model fit (calibration), and the model discriminatory ability was assessed via the Area Under the ROC (AUC).

## Results

### Identification and detection of *P. falciformis* in badger samples

**Fig. 1:**
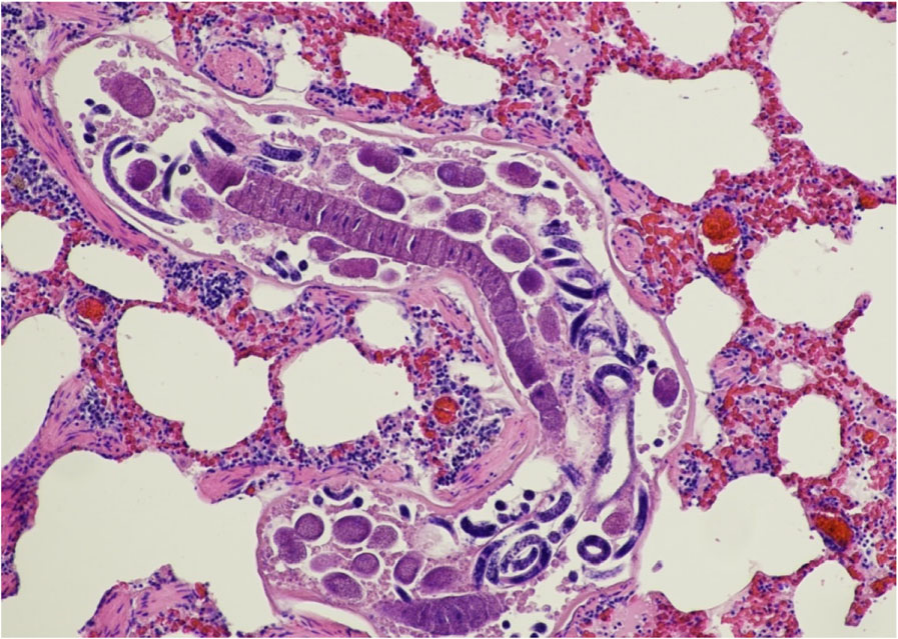
Longitudinal section through an adult *P. falciformis* containing larvae ( x 100)

Adult nematodes present in terminal airways were identfied as *P. falciformis* based on the morphological description provided by Davidson et.al. [11] allowing for correction for the body length from micrometres to millimetres. Identification was confirmed by two independent experts. The parasites were coiled, and cylindrical in outline with a smooth cuticle. Numerous adult parasites contained larvae and larvae were often free within the adult worm as opposed to being enclosed within an egg capsule (Fig. 1). Similar observations had been made by Inger Hamnes (personal communication). The width of the L1 larvae (10.5–11.9 μm) was within the range quoted for the species by Davidson et al. [11] and Deak et.al. [8]. Larvae or ova were not detected in the pulmonary arterial system. The size, pulmonary location and morphology of the parasites detected excluded *Crenosoma*, *Capillaria*, *Filaroides* and *Angiostrongylus* species.

Of the 15 badgers analysed by PCR in 2017, 12 were positive in at least 1 sample and 7 were positive in all 3 loci. All PCR products were 100% identical to each other and to the rRNA sequences and internal transcribes space are logged in the data base for *P. falciformis* accession number (KY365435). The badgers that tested positive for *P. falciformis* in this subset were located in Kilkenny (1/1), Kildare (4/4), Laois (1/1), Offaly (1/1) and Tipperary (5/6). The badgers sampled from Carlow (0/1) and Wexford (0/1) were negative. No other lung- or heartworm species were detected using molecular techniques.

**Fig. 2:**
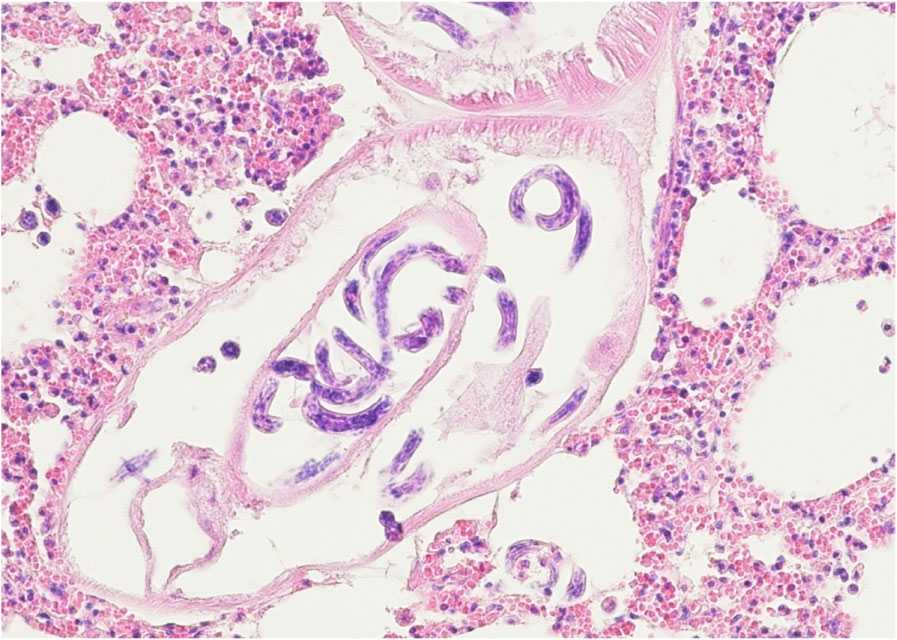
Cross section through an *P. falciformis* adult female containing larvae. Tissue reaction is not marked (× 200)

Pulmonary tissue sections positive for *P. falciformis* were never positive for typical bTB lesions. The tissue reaction to the parasite was not excessive, often minimal, and was not dominated by eosinophils (Fig. 2).

### Epidemiological findings

**Fig. 3:**
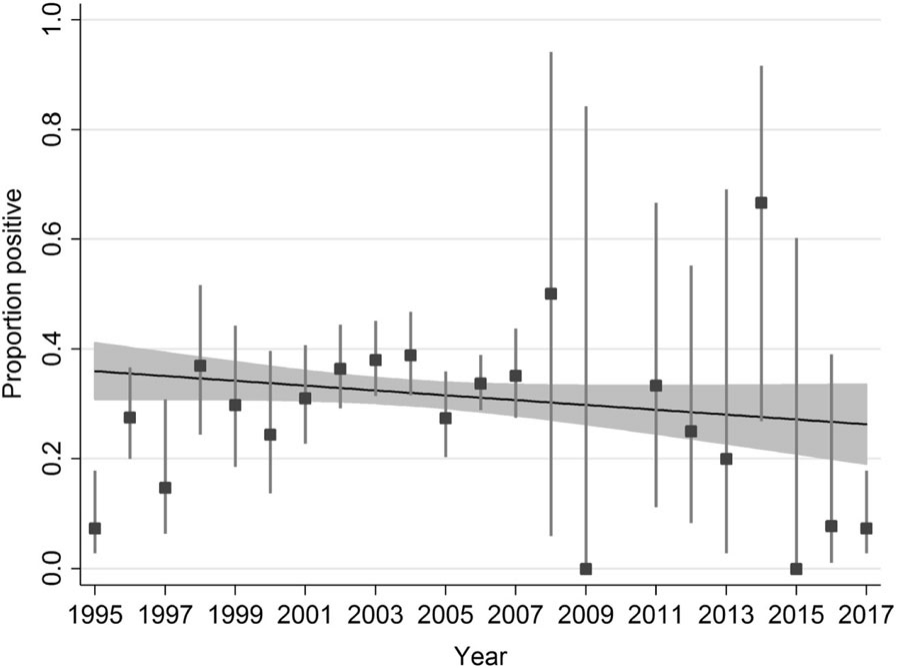
The estimated probability of infestation for each year of the time series. Point estimates were estimated from a logistic regression with error bars representing 95% CI, with the exception of years 2009 and 2015 which had zero prevalence, with upper 95% CI estimated using a one-tailed exact binomial CI. The line represents the estimated linear trend with 95% CI

Overall, there were 1580 badgers with a lungworm status, with an estimated prevalence of 32.09% (95% CI: 29.79–34.45%). There was significant inter-annual variability in prevalence (logistic regression with year fitted as categorical; LR χ^2^ (df: 18) = 46.35; *p* = 0.0003; Fig. 3). A linear trend fitted to the data suggested a non-significant decline in prevalence across the time series (OR: 0.980; *P* = 0.100).

**Fig. 4:**
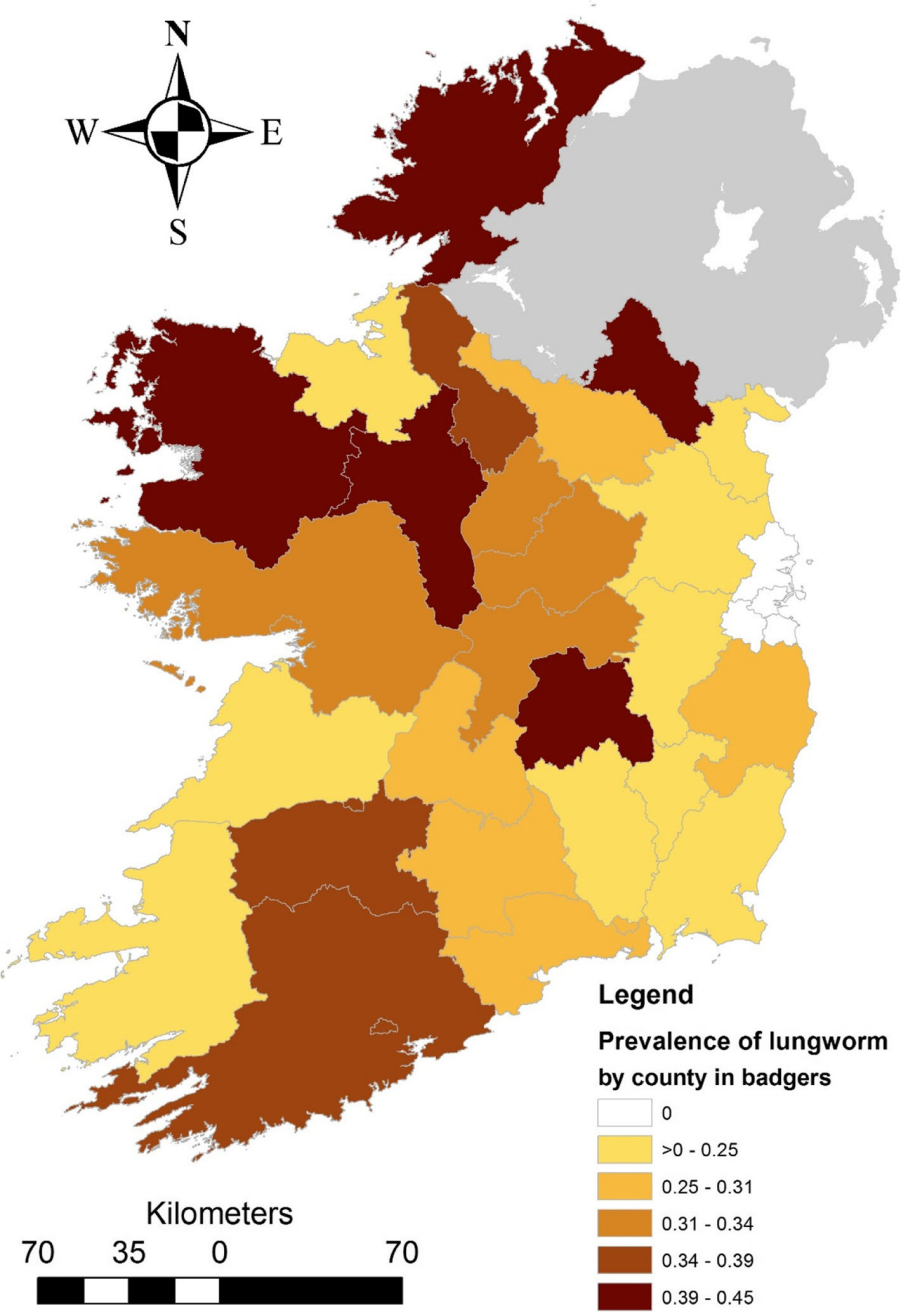
Choropleth map of the variation in prevalence of *P. falciformis* at County level across the Republic of Ireland. Note, Northern Ireland (greyed area) was not sampled for the current study

**Fig. 5:**
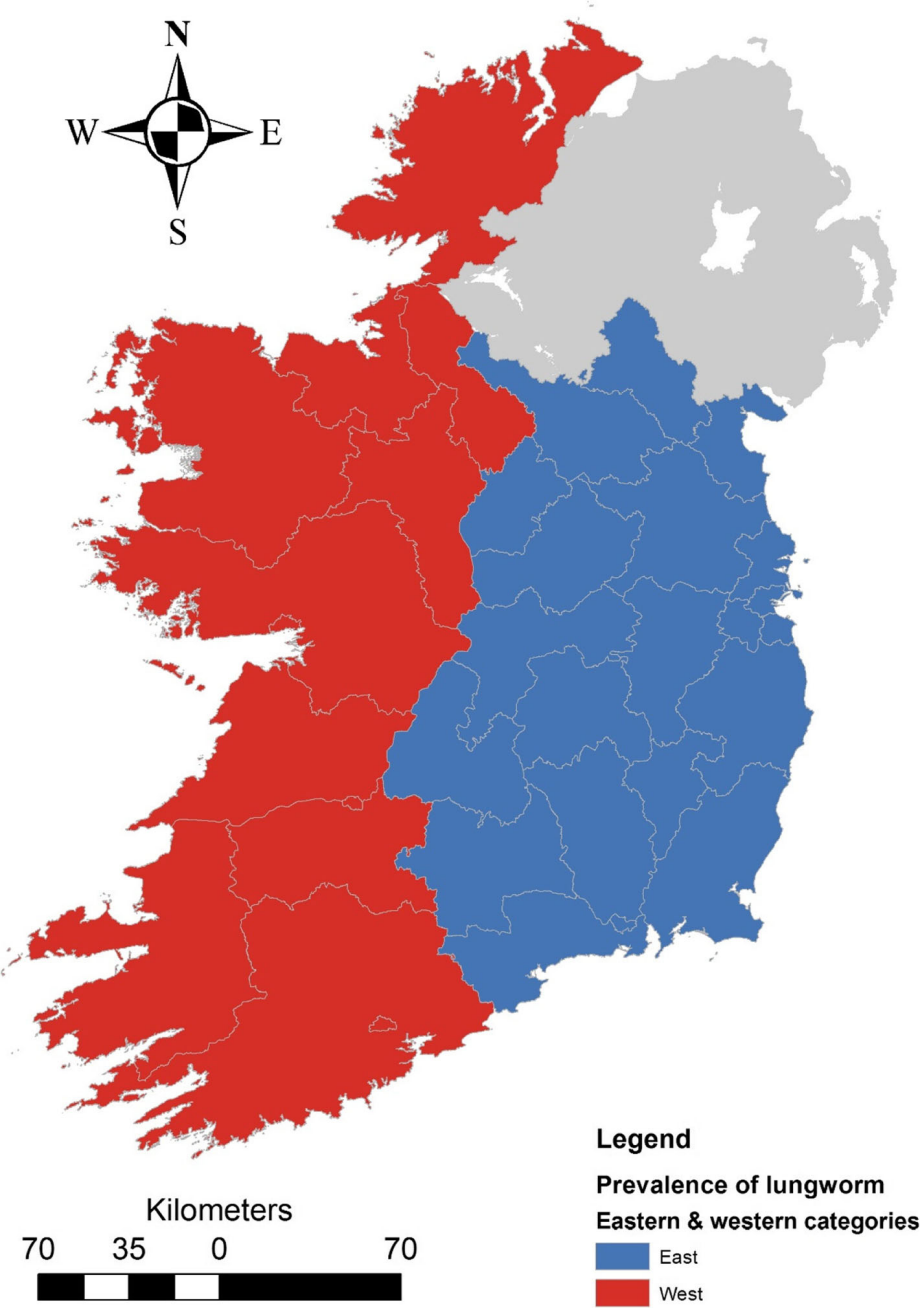
Supplementary material: Eastern versus western counties categorization used in final multivariable model

There were 49 records with some covariate data missing on archival data retrieval (e.g. 38 animals did not have a county assigned; 36 animals did not have a recorded sex), meaning there was a total of 1539 animal records available for modelling. There was significant variation in infestation risk across seasons, with infestation risk being higher in spring (OR: 1.443; *P* = 0.003; 95% CI: 1.135–1.835) and autumn (1.508; *P* = 0.008; 95% CI: 1.114–2.039) relative to animals caught in winter. There was no significant difference in risk between summer and winter (*p* = 0.535), however very few animals were captured during summer months to inform this pattern (*n* = 24 caught in summer). At the univariable level, females had a significantly lower probability of being infested compared to males (OR: 0.692; *P* = 0.001; 95% CI: 0.558–0.858), with 28.1% of females being positive relative to 36.1% of males. Adults (31.3%) were at a significantly lower risk of infestation relative to juveniles (48.2%) (OR: 0.491; *P* = 0.002; 95% CI: 0.315–0.765). There was significant variation in infestation risk by location (*p* = 0.001), with Mayo (45.0%; *n* = 100), Laois (42.9%; *n* = 42) and Offaly (42.9%; *n* = 241) having the highest levels of infestation, while Meath (9.7%; *n* = 31), Carlow (6.7%; *n* = 15) and Dublin (0%; *n* = 10) had the lowest prevalence. There was no strong geographic pattern of risk across the counties of the Republic of Ireland (Fig. 4), but prevalence appeared higher overall in western counties than eastern counties (Fig. 5).

During multivariable model building we aggregated both the year (*n* = 22 years) and county (*n* = 26 counties) variables to reduce the number of parameters in the model. We fitted year as a linear trend, a quadratic form, a logarithm transform and as a binary dummy variable (pre- and post-2007) in alternative models. We retained the binary categorization as it had the lowest AIC across comparative models (ΔAIC = 10). Given the pattern of prevalence across counties, we fitted a binary model for eastern (Dublin, Louth, Monaghan, Wicklow, Carlow, Kilkenny, Wexford, Waterford, Meath, Westmeath, Longford, Cavan Offaly and Laois) and western (Donegal, Sligo, Mayo, Galway, Roscommon, Clare, Leitrim, Limerick, Cork and Kerry) counties (see supplementary material for map). The final multivariable model is presented in Table 3; the AIC of the final model was 1886, with the next best model having an AIC of 1890. The AUC of the model suggested fair discriminatory ability at 0.62; there was no evidence for misspecification (calibration) in the model fit (Hosmer–Lemeshow test: *p* = 0.855). Overall, there was evidence of decreased risk for adult, female badgers, captured after 2007, relative to juvenile, males and badgers captured during or prior to 2007. Furthermore, there was increased risk of observing infestation in Spring and Autumn relative to Winter months, and increased risk in western counties relative to eastern and midland counties (Table 3).

**Table 3:** Multivariable logistic model assessing the relationship between badger lungworm infestation risk and animal, temporal, and spatial characteristics

Outcome: Infection status		Odds Ratio	Std. Err.	P > z	Lower 95%CI	Upper 95%CI
Age-class	Juvenile	1.000				
	Adult	0.456	0.107	0.001	0.288	0.722
Sex	Male	1.000				
	Female	0.659	0.074	0.000	0.528	0.822
Year	Year ≤2007	1.000				
	Year > 2007	0.338	0.097	0.000	0.192	0.595
Season	Winter	1.000				
	Spring	1.497	0.188	0.001	1.171	1.914
	Summer	0.739	0.393	0.570	0.261	2.095
	Autumn	1.706	0.274	0.001	1.245	2.336
Location	Eastern county	1.000				
	Western county	1.385	0.157	0.004	1.110	1.729
	Constant	0.870	0.220	0.580	0.530	1.427

## Discussion

Byrne and colleagues [17] were the first to report the presence of *P. falciformis* (still referred to as *A. falciformis*) in Irish badgers. Their study is chiefly based on the morphological identification of adult worms and larvae detected in heart, lungs and faecal samples. While this is considered a reliable method for the identifying adult worms first stage larvae of *P.falciformis* are morphologically very similar to those of *Angiostronglyus* species with which they may be confused [11]. The recent publication by Deak et al. [8] has provided a detailed morphiological description of the larvae and adults of *P. falciformis* as well as genetic information. The morphological and molecular data reported in our study agree with their findings and confirm the presence of *P. falciformis* in Irish badgers. We also provide a model of the geographic distribution *P. falciformis* in Ireland. The same species of lungworm has been reported from badgers in many parts of Europe and Russia (10–12; 18–21; 28). In contrast, a different species, *A. pridhami* has been identified in Spanish badgers [15] (even though the identification is disputed by some [8]). This is interesting, given the genetic association between Spanish and Irish badgers. *A. pridhami* primarily infests American mink, and has been identified in invasive American mink in Spain. Morphological and PCR analysis of additional badgers, particularly from the west and south-west of Ireland are required to establish whether there are other badger lungworm species present in Ireland.

In the present study no additional lung- or heartworms were detected. The reason for this may have been that we focused chiefly on the dorsal areas of the lung lobes, which may have precluded detection of other parasite species. Byrne et al. [17] reported *Crenosoma spp* infestation at a prevalence of 1% in their Irish badger sample. Several European publications have also reported both *Angiostrongylus vasorum* and *Angiostrongylus daskalovi* in badgers [6, 10, 15, 24, 25]. Neither parasite was detected in this study or by Byrne et al. [17] even though *A. vasorum* is an important parasite of dogs in Ireland and was detected in 39.9% of Irish red foxes (*Vulpes vulpes*) in a recent study [26]. Foxes and badgers can co-habit in badger sets and would have a similar environmental exposure [1, 27]. It is also noteworthy that trematodes or cestodes were not identified by Byrne et al. [17], despite being specifically looked for.

The prevalence of *P. falciformis* varies between studies. In Britain during routine examination of badgers for bTB just 1 out of 118 badgers was recorded as being infested with *P. falciformis* [18]. Other studies showed higher prevalence rates. In an Italian study 53% (10/19) badgers were found to be infested [10]; in a Norwegian study 56% (5/9) [11]; in Poland 22% (2/9) [12]. In Bosnia Herzegovina 100% (1/1) [14], Romania 33% (9/27) [8] and Germany 75% (3/4) of badgers were infested [8]. A study from Spain suggested that 6.4% of 47 badgers were infested with *A. pridhami* [15], while in a sample of 163 badgers in Portugal no lungworm were detected [26].

In this study, an estimated prevalence of 32.09% (95% CI: 29.79–34.45%) was found, with some evidence of a declining prevalence over the sampling period (1995 to 2017). While this may be a real decrease it could also be due to the reduced sampling protocol as described above. Byrne et al. [17] detected a 20% prevalence of *P. falciformis* in 289 Irish badgers between December 2016 and October 2017. It is also important to note that PCR analysis indicated a much higher infection rate (around 80%), however this figure is based on a very small subsample.

*P. falciformis* require gastropod intermediate hosts such as slugs and snails to complete their life cycle [8]. It is possible that mice and other small vertebrates for example frogs may act as auxiliary hosts, as for *A. pridhami*. Ireland would be deemed favourable for the survival of slugs, snails and frogs. In fact dietary investigation by Cleary [18] on a subsection of this population showed a high intake of frogs by the Irish badger which may increase the exposure and possible prevalence of this parasite.

We found significant county level variation in infestation risk, and a trend toward higher infestation rates in the western part of Ireland relative to the eastern counties, which could be related to weather conditions, as western counties have significantly higher rainfall and probably also a greater availability of intermediate/ axillary hosts than eastern counties.

During the present study, we found that males exhibited higher risk of infestation relative to females, and juveniles relative to adult badgers. The Spanish study noted no sex- or age-specific differences in prevalence rates with regard to *A. pridhami* [15]. The capture method in Ireland favours adult badgers [28], and consequently there was a lower number of juveniles included in this study. However, we still found a significantly higher prevalence in young badgers suggesting an age-prevalence relationship, perhaps indicating that mature animals develop a degree of resistance. *P. falciformis* females are ovoviviparous and lay embryonated eggs. Embryonated eggs are illustrated in the studies by Demiasszkiewicz et al. [12] and Deak et. Al. [8]. Free larvae within the adult worm have also been observed by Inger Hamnes (personal communication) and possibly represent hatched larvae in the post sampling period. It is interesting to note that in this study the badger immune reaction to *P. falciformis* could not be deemed excessive and was not dominated by eosinophils. Moreover no concurrent infections with *M. bovis* were observed in the sections examined. Similarly no association was found by Byrne et al. [17] between TB status and *P. falciformis* infestation. Parasitic infestations have been associated with modification of the host immune response to concurrent bacterial infections and allergic reactions, and there is on-going research into parasite –bTB coinfestation dynamics [19, 29].

While pathological studies have described and attributed serious pulmonary pathology and verminous pneumonia to the presence of this parasite [8, 14], this would not appear to be the case in this population. Coughing is a feature of heavy experimental infestations [8]. It is interesting to note that field operatives never report coughing in captured badgers. However, it is difficult to assign clinical signs to a wildlife host, as they are not normally seen in a clinical context.

## Conclusion

During the present study we have confirmed the presence of *P. falciformis* in Irish badgers using comparative morphological investigation and PCR. A large, long-term surveillance dataset indicates that lungworm infestation in badgers in Ireland is prevalent in the population at a level of 32.09% (95% CI: 29.79–34.45%) and widely distributed geographically. The analysis suggested significant geographical variation in infestation risk across counties, with a trend towards higher risk in western versus eastern counties of Ireland. At the animal level, males were at higher risk of infestation than females, and juveniles were at significantly higher risk of infestation than adult badgers. The data also contributes to an understanding of the complexities of the host/parasite interaction.

## Data Availability

Animals were not specifically killed for this study. The animals were provided by the Irish Department of Agriculture, Food and the Marine (DAFM).
